# Winter peaks in web-based public inquiry into epistaxis

**DOI:** 10.1007/s00405-020-05915-x

**Published:** 2020-03-16

**Authors:** David Tianxiang Liu, Gerold Besser, Thomas Parzefall, Dominik Riss, Christian A. Mueller

**Affiliations:** Department of Otorhinolaryngology, Head and Neck Surgery, Medical University of Vienna, Währinger Gürtel 18-20, 1090 Vienna, Austria

**Keywords:** Epistaxis, Nosebleed, Seasonality, Google trends, Cosinor, Infodemiology

## Abstract

**Purpose:**

Epistaxis represents the most frequent ear, nose, throat-related emergency symptom. Seasonal variation in epistaxis incidence, with peaks during winter months, is widely accepted, although the literature itself remains inconclusive. The objective of this study was to evaluate public inquiry into nose bleeding, by considering Google-based search query frequency on “Epistaxis”-related search terms and to assess possible seasonal variations globally.

**Methods:**

Epistaxis-related search terms were systematically collected and compared using Google Trends (GT). Relative search volumes for the most relevant epistaxis-related terms, covering a timeframe from 2004 to 2019 were analysed using cosinor time series analysis for the United States of America, Germany, the United Kingdom, Italy, Canada, Australia, and New Zealand.

**Results:**

Graphical representation revealed seasonal variations with peaks during winter months in the majority of countries included. Subsequent cosinor analysis revealed these variations to be significant (all *p* < 0.001).

**Conclusion:**

Public interest in seeking epistaxis-related information through the Internet displayed seasonal patterns in countries from both hemispheres, with the highest interest during winter months. Further studies exploring causality with environmental factors are warranted.

**Electronic supplementary material:**

The online version of this article (10.1007/s00405-020-05915-x) contains supplementary material, which is available to authorized users.

## Introduction

Epistaxis, commonly known as nosebleed, is the most frequent ear, nose, throat (ENT)-related emergency symptom [1]. Simple first aid intervention (e.g., compression) can stop acute hemorrhage in the majority of cases, resulting in epistaxis being a self-limiting symptom with only 10% of patients seeking medical help [2]. Indeed, it is a common belief that meteorological factors such as air temperature and humidity affect the incidence of epistaxis, with maximal and minimal incidences occurring during the winter and summer months, respectively [1, 3–8]. However, the scientific literature on this topic is inconclusive. While some studies showed no association between seasonal variation and incidence [4, 9–11], others have shown the opposite pattern of results [1, 3, 5–8]. These open issues relate not only to the seasonality of epistaxis incidence, but importantly also to the validity and timing of online educational measures.

In recent years, for better or worse, the World Wide Web has become one of the most important sources of information available to the general population [12]. A report from 2013 showed that no less than 72% of all internet users have already searched for health-related issues. Particularly worthy of mention is Google web search, which represents the most commonly used search engine worldwide and provides the data foundation for further analysis [13]. A popular tool for big data mining is represented by Google Trends (GT), which allows users to explore oscillations in public interest, by selecting filters on geographical location and time [14]. Owing to these circumstances, it is not surprising that many health-related issues have already been associated with internet-search peaks in recent years to complement traditional data sources [15–22]. Noteworthy, previous studies on nose bleeding and GT suffered from two major methodological shortcomings: First, no systematical approach was chosen for evaluating epistaxis-related search terms that are representative for the general population. Cervellin [12] and Braun et al. [23] used the medical terms [*epistaxis*] and [*epistassi*], rather than the country-specific non-medical term [*nasenbluten*] or [*sangue dal naso*] as primary search terms. Secondly, the validity of the conclusions was compromised due to the absence of hypothesis testing. Braun et al. [23] investigated the search inquiry for epistaxis-related search terms in Germany, but provided only graphical evidence. Given these limitations, there is currently no clear indication about seasonal variations of public interest in epistaxis-related search terms on a worldwide basis.

Therefore, the aim of this study was to utilize Google Trends to test our hypothesis that search query frequencies of relevant epistaxis-related terms show peaks during winter months, independent from the hemisphere. Additionally, extracted GT data were assessed for validity and reproducibility, particularly in countries with fewer inhabitants. Lastly, country-specific search query frequency was compared with ambient weather factors. The results are discussed in terms of the validity of internet tools to predict peaks in information demand for nosebleeds, and of the measures needed to guide patients to reliable medical tips for stopping epistaxis.

## Material and methods

### Google Trends

Google Trends (Google Inc. [14]) is a dynamic and publicly accessible, internet-based search term analysis tool that allows assessment of search query frequency of terms and keywords queried on Google Web Search, which represents the most popular search engine worldwide [13]. Search queries can be specified by geographical location (e.g. country or state), timeframe (real time or non-real time/customized timeframe, dating back to January 2004), category (e.g. health or sports), and content (e.g. web search or news search).

GT displays the search query frequency as relative search volume (RSV), which is a normalized value to allow comparability between different terms (up to five). The process is described in detail elsewhere [24]. Briefly, GT calculates the number of searches done for a particular term divided by all searches done using Google search engine adjusted to location and time, to avoid a possible bias of total search volume, as otherwise countries with the highest total search volume would also position highest. In a next step, these numbers are rescaled and displayed as whole numbers ranging from 0 to 100 (relative search volume, RSV), relating to the proportion of a specific topic compared to all search queries on all topics.

Further, GT provides the feature "related queries", which displays similar search terms entered by other users during searches for the specified one. Depending on timeframe specification, results are then displayed either in the “Top” (meaning the most popular search queries) or “Rising” (queries with biggest increase in frequency since the last time period) category. Additionally, GT also excludes duplicate searches from the same user within a short timescale.

As already noticed by other authors [25], search queries on GT using fixed filters, done on different days (and even different minutes) lead to (slightly) different results during each new search query, because (apparently) a different “random sample” from all search queries done on Google Web search is selected each time. Interestingly, data queried on different timepoints from countries with large population sizes proved a rather high reliability, whereas data from smaller countries were much less reliable [25].

### Search strategy

#### Geographical location

Similar to previous works on seasonal variations of health-related issues [16–19, 26, 27], the authors specified English and non-English-speaking countries from both hemispheres as geographical location for further analysis: Australia, Canada, Germany, Italy, New Zealand, Norway, the United Kingdom (UK), and the United States of America (USA). The first step included the evaluation of country-specific, epistaxis related search terms, since we included countries with different official languages. Therefore, results from team brainstorming by all authors and search terms from previous studies on GT and epistaxis [12, 23] were compiled together and noted as primary search terms (Table 1).

**Table 1:** Primary search terms (previously published and brainstorming) and the respective search terms with the highest relative search volume, which were subsequently selected for further analyses

Country	Primary search terms	Most relevant^b^
English-speaking countries^a^	“nosebleed”, “nose bleeds”, “nose bleed”, “nosebleeds”, “epistaxis”	[nose bleeds]
Germany	“nasenbluten”, “epistaxis”	[nasenbluten]
Italy	“sangue da naso”, “sangue dal naso”, “epistassis”, “epistaxis”	[sangue dal naso]
Norway	“neseblod”, “epistaxis”	[neseblod]

^a^English speaking countries: Australia, Canada, New Zealand, United Kingdom, United States of America

^b^Search term with the country-specific, highest relative search volume

#### Timeframe

All GT queries were specified for the timeframe between January 1, 2004 and September 30, 2019, to cover the longest timeframe of GT-records available. Furthermore, the “health” category was selected to evaluate health specific interest. In accordance to previously published studies, winter months were defined as December, January, February and March for the northern hemisphere, whereas June, July, August and September were assigned to the southern hemisphere [15–17, 28]. The present study followed the checklist for use of GT in health care research by Nuti et al. [21].

#### Country-specific epistaxis-related search terms

GT was assessed on October 20, 2019 and the country-specific, most representative (most relevant) search terms were selected using a systematic, stepwise approach [25]. Firstly, GT function “related queries” was used to add further, epistaxis-related search terms to the primary search terms (Supplement Table 1). Secondly, all previously collected search terms were then compared with each other using GT comparison function. As GT only allows the comparison of 5 search terms simultaneously, the term with the highest RSV always remained unchanged and other search terms were replaced gradually. This represented a crucial step, as we wanted to conduct further analysis with the country-specific highest epistaxis-related search term to avoid Type I related errors as a result of non-representative or insufficient RSV.

### Validity and reproducibility of GT data

The second step included the assessment of validity and reproducibility of GT data queried at different time points, using the country-specific, most relevant search terms. Therefore, daily inquiries for monthly data were made from October 20, 2019 until October 27, 2019. Comma-separated value files (CSV) were downloaded on above-mentioned eight consecutive days, resulting in 189 data points for each country and day (15 years × 12 months + 9 months). Daily data represented single/individual time series data, whereas averaged time series data of individual countries were calculated as the mean of data queried on above mentioned 8 days.

### Population data

To assess a possible correlation between population size and reliability of time series data, number of inhabitants (in million [29]) from included countries were also noted on October 27, 2019: Australia 25.31; Canada 37.53; Germany 83.62; Italy 60.54; New Zealand 4.80; Norway 5.39; United Kingdom 67.66; United States of America 329.74.

### Climate data

Climate data reported herein were retrieved from the Climate Data Center (CDC) of the German Weather Service (Deutscher Wetterdienst, DWD). It is a public institution and an agency of the German Federal Ministry of Transport and Digital Infrastructure (BMVI), which provides publicly accessible, web-based data. Meteorological data used during this study included monthly summaries, recorded by meteorological stations located in the cities of Berlin (Berlin-Tegel) and Munich (Munich-Airport), which represent the two most densely populated cities in Germany. We retrieved following parameters (monthly mean): temperature—air temperature 2 m above ground (°C), sunshine duration (hours), humidity—relative humidity at 2 m above ground (%), air pressure—station level (hPa), and vapor pressure (hPa). All climate data were assessed for the timeframe between January 1, 2004 and September 30, 2019, matching that of the GT-searches.

### Statistical analysis

Cosinor analysis was used to assess seasonal variations in RSV for above-mentioned time series and integrated into two steps: (a) data visualization and (b) model fitting. The operating system and methods are presented in detail elsewhere [30]. In short, cosinor analysis is a parametric seasonal model that fits a sine wave to a predefined timeframe as part of the generalized linear model and computes following variables: amplitude (size) *A* and phase (peak) *p*, based on length (duration) c defined as 12 (annual seasonal cycle = 12 months). Therefore, the peak is defined once a year and the low point (nadir) *L* is calculated as peak ± 6 (months). Since both sine and cosine functions characterize the seasonal variation within the cosinor model, statistical significance can be tested as part of the generalized linear model with *p* value set at 0.025 to adjust for multiple comparisons. Subsequently, to quantify reliability of individual and averaged time series data, intraclass correlation coefficients using the two-way-random model were performed according to Shrout and Fleiss [31]. Bivariate correlation was calculated using Spearman rank correlation (*r*_s_). Data analysis and visualization were carried out using the “season” and “psych” package in R 3.5.1 (R Development Core Team, 2008; R Foundation for Statistical Computing, Vienna, Austria) and Graph Pad Prism 8.2 (GraphPad Software, Inc., La Jolla, CA).

## Results

### Averaged time series data analysis shows peaks during winter months

To determine whether Google Trends might pick up seasonal patterns in searches for nosebleed, we performed our analysis using averaged time series data queried on eight consecutive days for eight countries representing both hemispheres (listed below), as search queries done on different days were not absolutely identical during our searches. To include the longest period of GT records available, we entered our searches beginning with 2004.

The results of the seasonality analysis revealed significant seasonal patterns and peaks during winter in Australia (*A* = 6.3, *P* = 8.7, *p* < 0.001), USA (*A* = 10, *P* = 2.3, *p* < 0.001), and Canada (*A* = 10.9, *P* = 2.6, *p* < 0.001). The same pattern was found to be statistically significant in the United Kingdom (*A* = 12.6, *P* = 3, *p* < 0.001), Germany (*A* = 13.4, *P* = 2.7, *p* < 0.001), Norway (*A* = 2.5, *P* = 2.8, *p* < 0.001), and Italy (*A* = 10.5, *P* = 3.1, *p* < 0.001). Only New Zealand showed a slightly shifted peak in October (*A* = 2.5, *P* = 10, *p* < 0.001; all Table 2).

**Table 2:** Cosinor analysis on seasonality of epistaxis related search terms

Country	Time series	Amplitude	Peak^a^	Nadir^a^	Standard error	*p *value
Australia	Single	5.2	9.1	3.1	0.018	< 0.001
	Average	6.3	8.7	2.7	0.017	< 0.001
Canada	Single	15.8	2.4	8.4	0.012	< 0.001
	Average	10.9	2.6	8.6	0.013	< 0.001
Germany	Single	13.4	2.7	8.7	0.015	< 0.001
	Average	13.4	2.7	8.7	0.015	< 0.001
Italy	Single	12.1	3.1	9.1	0.016	< 0.001
	Average	10.5	3.1	9.1	0.016	< 0.001
New Zealand	Single	1.6	1.6	7.6	0.028	< 0.001
	Average	2.5	10	4	0.024	< 0.001
Norway	Single	3.9	2.1	8.1	0.014	< 0.001
	Average	2.5	2.8	8.8	0.021	< 0.001
United Kingdom	Single	12.9	3.1	9.1	0.014	< 0.001
	Average	12.6	3	9	0.014	< 0.001
United States of America	Single	10.5	2.3	8.3	0.012	< 0.001
	Average	10	2.3	8.3	0.012	< 0.001

*Single* individual time series, *Average* averaged time series

^a^The number represents the corresponding month as follows: 1 = January, 2 = February, 3 = March, 4 = April, 5 = May, 6 = June, 7 = July, 8 = August, 9 = September, 10 = October, 11 = November, 12 = December

Hence, it would seem that GT averaged time series data showed winter peaks for nosebleed in the majority of the countries included, which was in accordance with our primary hypothesis. In a next step, seasonality analysis was performed using single time series data queried on the first day of data extraction, October 20, 2019.

### Single time series data analysis shows peaks during winter months

**Fig. 1:**
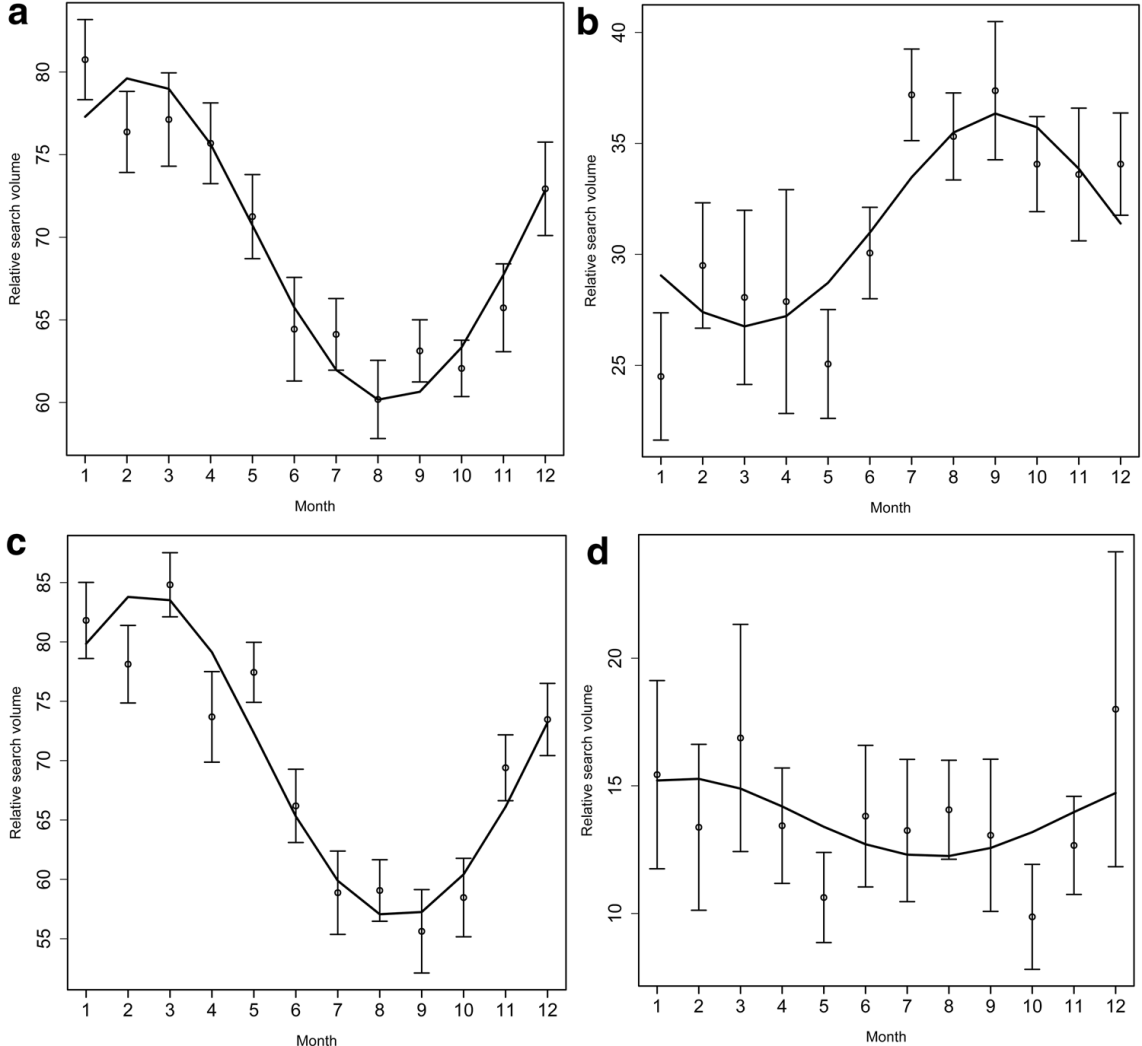
Seasonal variation for epistaxis related search terms [nose bleeds] in the United States (**a**), Australia (**b**), Canada (**c**), and New Zealand (**d**) from January 1, 2004 to September 30, 2019. The curves represent the adjusted cosinor analysis model. Points represent monthly means and the two horizontal lines mark the standard error. 1 = January, 2 = February, 3 = March, 4 = April, 5 = May, 6 = June, 7 = July, 8 = August, 9 = September, 10 = October, 11 = November, 12 = December

**Fig. 2:**
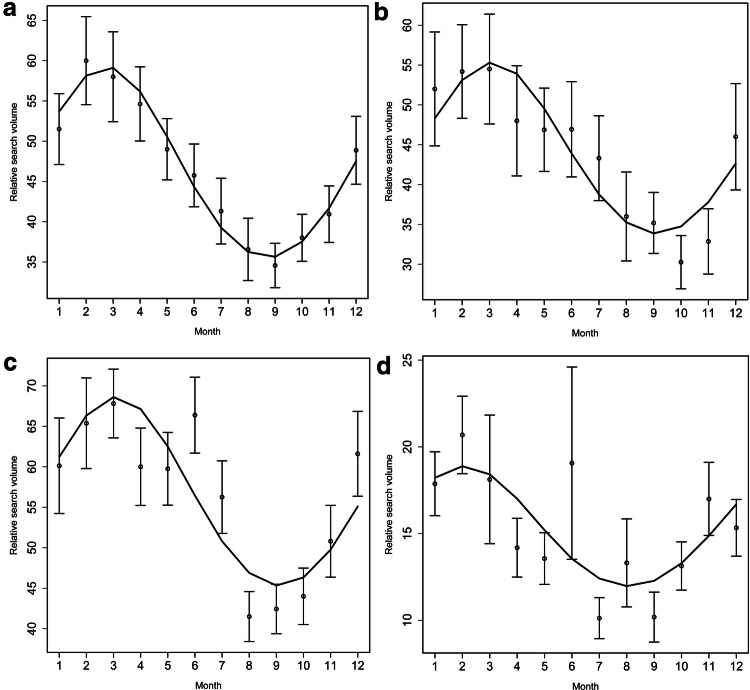
Seasonal variation for epistaxis related search terms [nasenbluten] in Germany (**a**), [sangue dal naso] in Italy (**b**), [nose bleeds] in the United Kingdom (**c**), and [neseblod] in Norway (**d**) from January 1, 2004 to September 30, 2019. The curves represent the adjusted cosinor analysis model. Points represent monthly means and the two horizontal lines mark the standard error. 1 = January, 2 = February, 3 = March, 4 = April, 5 = May, 6 = June, 7 = July, 8 = August, 9 = September, 10 = October, 11 = November, 12 = December

Seasonality analysis using single time series data revealed seasonal patterns during the winter months in Australia (*A* = 5.2, *P* = 9.1, *p* < 0.001), USA (*A* = 10.5, *P* = 2.3, *p* < 0.001), and Canada (*A* = 15.8, *P* = 2.4, *p* < 0.001). Similarly, significant patterns were obtained for the United Kingdom (*A* = 12.9, *P* = 2.3, *p* < 0.001), Germany (*A* = 13.4, *P* = 2.7, *p* < 0.001), Norway (*A* = 3.9, *P* = 2.1, *p* < 0.001), and Italy (*A* = 12.1, *P* = 3.1, *p* < 0.001). Again, New Zealand was the only country that underwent a change in seasonal peak (*A* = 1.6, *P* = 1.6, *p* < 0.001; all Table 2). Graphical visualization and fitted sinus waves are presented in Figs. 1 and 2.

Therefore, with the exception of New Zealand, GT single time series data showed peaks during the winter months and, thus, it seemed interesting to determine the reliability of GT at exposing patterns in searches for nosebleed, but also to assess whether this reliability might be dependent on population sizes.

### Strong correlation between population size and reliability of single time series data

We calculated the reliability of GT by determining intra-class correlation coefficients of single time series data for included countries queried over eight consecutive days, as mentioned above. In a next step, we performed correlation analysis using Spearman’s correlation coefficient so as to resolve the association between reliability of single time series data and the number of inhabitants.

Both single and average intra-class correlations of each country were highly significant (*p* < 0.0001), but the averaged seasonal data demonstrated higher reliabilities than individual data, as seen from larger correlation coefficients (Table 3). Further analysis revealed a strong correlation between reliability of single time series data and population size (*r*_s_ = 0.81, *p* < 0.005, Spearman correlation).

**Table 3:** Reliability of Google Trends individual and averaged time series data on nosebleed related search terms presented as intraclass correlation coefficient using the two-way random model

Country	Time series	Correlation coefficient	Lower bound	Upper bound	*F*	*Df1*	*Df2*	*p *value
Australia	Single	0.40	0.29	0.50	10.9	188	1504	< 0.0001
	Average	0.86	0.78	0.90	10.9	188	1504	< 0.0001
Canada	Single	0.49	0.38	0.59	14.4	188	1504	< 0.0001
	Average	0.90	0.85	0.93	14.4	188	1504	< 0.0001
Germany	Single	0.98	0.97	0.98	427	188	1504	< 0.0001
	Average	1.00	1.00	1.00	427	188	1504	< 0.0001
Italy	Single	0.96	0.95	0.97	238	188	1504	< 0.0001
	Average	1.00	0.99	1.00	238	188	1504	< 0.0001
New Zealand	Single	0.31	0.25	0.38	5.8	188	1504	< 0.0001
	Average	0.80	0.75	0.85	5.8	188	1504	< 0.0001
Norway	Single	0.47	0.39	0.55	11.5	188	1504	< 0.0001
	Average	0.89	0.85	0.92	11.5	188	1504	< 0.0001
United Kingdom	Single	0.92	0.90	0.94	133	188	1504	< 0.0001
	Average	0.99	0.99	0.99	133	188	1504	< 0.0001
United States of America	Single	0.90	0.88	0.92	96	188	1504	< 0.0001
	Average	0.99	0.98	0.99	96	188	1504	< 0.0001

*Single* individual time series, *Average* averaged time series, *Lower and Upper bound* 95% confidence interval, *FF* test for the significance of correlation, *Df1* numerator degrees of freedom, *Df2* denominator degrees of freedom

### Weak correlation between temperature and relative search volume for epistaxis-related search terms

To assess whether ambient weather factors correlate with relative search volume of epistaxis related search terms, we performed bivariate correlation analysis using meteorological data retrieved from weather stations located at two of the most densely populated cities (Berlin and Munich) and averaged time series data from Germany.

The results from correlation analysis revealed a weak correlation between temperature and vapor pressure with averaged time series data (Table 4). No relevant correlation was found between sunshine, humidity, air pressure, and relative search volume.

**Table 4:** Bivariate correlation between averaged time series data on nosebleed related search terms and ambient weather factors in Munich and Berlin between 2004 and 2019

		Temperature	Sunshine	Humidity	Air pressure	Vapor pressure
Munich	*r*_s_	− 0.22*	− 0.06	− 0.04	− 0.01	− 0.28***
Berlin	*r*_s_	− 0.23*	− 0.07	− 0.06	0.13	− 0.33***

*r*_*s*_ correlation coefficient

*p < 0.05

***p < 0.001

## Discussion

The vast majority of epistaxis cases can be handled with simple measurements. At the same time, epistaxis represents a very frequent symptom in outpatient emergency departments that might also require acute intervention. Although it is generally assumed (and even taught) at university level that the incidence of epistaxis increases during winter months, the literature itself remains contradictory. In this regard, Google Trends has become a valuable tool to expand existing knowledge and provide further information for hypothesis formulation [21, 22]. Here, we showed for the first-time seasonal differences in Google search query frequency for epistaxis-related search terms in various countries, from both hemispheres, indicating that the search query frequency for epistaxis-related search terms follow a seasonal pattern. In addition, these differences were also evident in non-English-speaking countries, which supported our primary findings.

It should be emphasized that although our data showed seasonal variations in Google search volume for epistaxis related terms, the current results must be interpreted cautiously, as the nature of these data do not allow biological causalities to be determined. A general increase in overall medical inquiries during winter weather conditions (e.g., more time at home) may be a relevant confounding factor and has to be taken into consideration in future studies on seasonal differences in Google search volume for health-related issues [32]. However, the finding that Google search query frequency for epistaxis-related symptoms increased during the winter months was not altogether surprising. It has been reported previously that epistaxis is more frequent in winter weather conditions [1, 3–8, 33]. One possible cause is related to the dry and heated-, or cold, air during the winter months (indoor or ambient), which leads to nasal mucosa desiccation, making it friable and vulnerable. Interestingly, Cruz et al. [34] studied a subgroup of patients that were sensitive to cold and dry air (e.g., having typical rhinitis-associated symptoms such as a congested nose during winter months). It was found that nasal challenge with cold dry air lead to a statistically significant higher count of epithelial cells being shed away (“epithelial shedding”) compared to warm and moist air, thus providing evidence for the mucosa-desiccation theory in this subgroup of patients with non-allergic rhinitis.

In reference to the negative correlation between temperature and search volume of epistaxis related search terms, this was commensurate with previous results relating to meteorological factors and incidence of epistaxis in adults presenting to an outpatient emergency clinic. Comelli et al. [7] identified a strong, negative correlation for temperature, and a moderate, negative correlation for humidity and epistaxis incidence. Similarly, Sowersby et al. [8] also found a strong, negative correlation for ambient temperature, although no correlation was found between humidity and epistaxis incidence. On the other hand, Reddy et al. [4] reported no relevant correlation between daily incidence of epistaxis and weather variables including ambient temperature, atmospheric pressure, and water vapour pressure. Interestingly, we found a negative correlation between vapor pressure and search volume of epistaxis related search terms. It is not yet possible to explain the reason behind this relationship, however, this finding might serve as a novel reference point from which to consider further studies on epistaxis incidence and vapor pressure. Based on current data, the association between meteorological variables and epistaxis incidence can only be speculated upon.

Considering the direct correlation of air temperature and incidence of respiratory tract infection of the upper airway (e.g. “common cold”) it is not surprising that viral infections have already been linked to epistaxis [35–38]. Other factors that might lead to the higher incidence of epistaxis during the winter months which may overstrain the already stressed mucous membrane include: (i) changes at the cellular level, such as the disruption of the epithelial cell barrier, and (ii) the mechanical effect of increased sneezing or nose blowing when affected by cold [39].

First aid measures in cases of epistaxis had been described previously and outlined in detail by Tunkel et al. [40]. Another plausible explanation for the higher Google search volume in epistaxis-related search terms during the winter months may be related to the self-limiting nature of this condition, as it is assumed that only 10% of all cases will require medical assistance [2]. Considering that (i) internet penetration in the countries surveyed was among the highest in the world [41], (ii) 72% of the general population already searched for health-related information online [42], and (iii) Google represented the most popular search engine (~ 65% of the total volume [13]), it is tempting to speculate the existence of a linear correlation between search query frequency for epistaxis-related search terms and epistaxis incidence.

Regarding the issue of non-English-speaking countries, these also showed a significant seasonality with peaks during the winter months in Europe, which supported our primary hypothesis. A 2013 GT study using [*epistaxis*] as search term in Germany concluded that the seasonality of epistaxis was coincidental with the winter months [23]. Our present results supported this hypothesis, albeit we additionally demonstrated seasonality for the German word [*nasenbluten*], representing the epistaxis search term with the highest relative search volume in Germany, based on statistical methods.

The authors of a previous study on association between GT time series data of epistaxis related search terms [*sangue da naso*] and [*epistassi*] covering an Italian province of about ~ 450.000 inhabitants and real population epidemiology of epistaxis patients visiting the local emergency department concluded no overlap [12]. From the present perspective, a major factor for this conclusion may have been missed, namely a systematic approach for representative epistaxis-related search terms. Notably, [*sangue dal naso*] had a ~ 11-fold higher RSV compared to [*sangue da naso*] during our search queries. Additionally, we support the conclusion of Tran et al. [25] on the reliability of GT time series data retrieved from countries with smaller population sizes, as individual GT time series data were also less reliable in our study. Therefore, we also recommend assessing GT time series data on several days in a row and to verify the reliability of included data, and in cases of poor reliability, to use averaged time series data for analysis purposes. Interestingly, the country with the smallest population size included (New Zealand) also showed the poorest reliability of individual time series and, more importantly, was also the only country undergoing a change in seasonal peak when using averaged time series data, which supported this hypothesis.

Concerning the clinical relevance of this work, our results might have importance at an epidemiological level. Epistaxis requires immediate action by affected individuals, which raises important challenges, related to distribution of reliable and easy understandable advices. The potential danger of misinformation (i.e., the common misconception “Tilt the head back during epistaxis”) underlines the need to make greater efforts in web-based guiding and monitoring of health-information seeking individuals [43]. A further important point is reflected in timing for providing key facts and education to the general public about first aid measures, based on seasonality and incidence peaks. By efficiently distributing and disseminating first aid information among the population, this might reduce the number of hospital visits, ultimately leading to greater financial economy.

## Conclusion

Global public interest in seeking epistaxis-related information through the internet displayed seasonal patterns, with the highest interest peaking during winter months. This novel finding may provide further evidence for the seasonality of epistaxis incidence. However, additional clinical and experimental studies will be warranted to explore any causalities with obvious factors such as meteorological variables.

## Electronic supplementary material

Below is the link to the electronic supplementary material.

Supplementary file1 (DOCX 19 kb)
