# Supplementary material for: Winter peaks in web-based public inquiry into epistaxis
Source: Eur Arch Otorhinolaryngol. 2020 Mar 16;277(7):1977–85. doi: 10.1007/s00405-020-05915-x (PMC7286850; doi:10.1007/s00405-020-05915-x)
Supplement: Supplementary file 1 — Supplementary file1 (DOCX 19 kb) [file 405_2020_5915_MOESM1_ESM.docx]

**Supplement Table 1.** Country-specific search terms that were evaluated and compared with each other during this study.

**UNITES STATES OF AMERICA**

agoraphobic nosebleed, anime nosebleed, anterior epistaxis, bad nose bleed, bad nose bleeds, bloody nose, cause of nose bleed, cause of nose bleeds, cause of nosebleeds, causes for nosebleeds, causes of nose bleed, causes of nose bleeds, causes of nosebleeds, child nosebleeds, chronic nose bleeds, chronic nosebleeds, constant nose bleeds, constant nosebleeds, define epistaxis, definition of epistaxis, dry nose bleed, dry nose bleeds, epistasis, epistaxis causes, epistaxis definition, epistaxis icd 9 code, epistaxis medical definition, epistaxis medical term, epistaxis symptoms, epistaxis treatment, excessive nose bleeds, frequent nose bleeds, headaches and nosebleeds, high blood pressure nose bleed, high blood pressure nosebleeds, how to stop a nose bleed, how to stop a nosebleed, how to stop nosebleed, how to stop nosebleeds, icd 10 code for epistaxis, icd 10 epistaxis, icd 10 for epistaxis, nose bleed, kids nose bleed, kids nosebleeds, nose bleed cause, nose bleed causes, nose bleed stop, nose bleed treatment, nose bleeding, nose bleeds, nose bleeds cause, nose bleeds everyday, nose bleeds how to stop, nose bleeds in children, nose bleeds in kids, nosebleed, nosebleed and headache, nosebleed cause, nosebleed causes, nosebleed headache, nosebleed medical term, nosebleed pregnancy, nosebleed seats, nosebleed section, nosebleed stop, nosebleed symptoms, nosebleeds, nosebleeds cause, nosebleeds causes, nosebleeds in adults, nosebleeds in children, nosebleeds in kids, posterior epistaxis, pregnancy nosebleeds, prevent nosebleeds, random nose bleed, random nose bleeds, reasons for nosebleeds, severe nose bleed, severe nose bleeds, sinus nose bleed, sinus nose bleeds, stop a nose bleed, stop a nosebleed, stop nose bleeds, sudden nose bleed, what can cause nose bleeds, what cause nose bleed, what cause nose bleeds, what causes a nose bleed, what causes nose bleed, what causes nose bleeds, what causes nose to bleed, what causes nosebleed, what causes nosebleeds, what is epistaxis, what to do nosebleed, why nosebleed, why nosebleeds

**CANADA**

baby nose bleed, bleeding nose, bleeding nose, bleeding nose, cause of nosebleeds, causes of nosebleeds, chronic nosebleed, constant nose bleeds, constant nosebleeds, epistasis, epistaxis, epistaxis definition, epitaxis, frequent nose bleeds, frequent nosebleeds, how to stop a nose bleed, how to stop a nosebleed fast, how to stop a nosebleed, how to stop a nosebleed, how to stop nose bleed, how to stop nose bleed, how to stop nose bleed, how to stop nose bleeds, how to stop nosebleeds, leukemia, nose bleed, nose bleed cause, nose bleed cause, nose bleed causes, nose bleed causes, nose bleed for no reason, nose bleed in kids, nose bleed what to do, nose bleed, nose bleed, nose bleeds, nose bleeds cause, nose bleeds everyday, nose bleeds for no reason, nose bleeds in children, nose bleeds in kids, nose bleeds reasons, nose bleeds what to do, nose bleeds, nose bleeds, nose bleeds, nosebleed, nosebleed cause, nosebleed causes, nosebleed treatment, nosebleed what to do, nosebleed, nosebleed, nosebleeds, nosebleeds causes, nosebleeds in children, nosebleeds in kids, nosebleeds in winter, nosebleeds, nosebleeds, random nose bleed, random nose bleeds, stop nose bleed, thrombocytopenia, what causes nose bleeds, what causes nosebleeds, what causes nosebleeds

**AUSTRALIA**

bleeding nose, bleeding nose, bleeding nose, blood from nose, blood in mucus, blood in nose mucus, blood in nose, blood in phlegm, blood nose, blood nose cause, blood nose causes, blood nose first aid, blood nose pregnancy, blood nose treatment, blood nose what to do, blood nose, blood nose, blood noses, blood noses, bloody nose, causes of nosebleeds, constant nose bleeds, dry blood in nose, epistasis, epistaxis, epistaxis definition, epitaxis, frequent nose bleeds, frequent nosebleeds, horse nose bleed, how to stop a blood nose, how to stop a nose bleed, how to stop a nosebleed, how to stop blood nose, how to stop nose bleed, how to stop nose bleeds, how to stop nosebleeds, nose bleed, nose bleed cause, nose bleed causes, nose bleed, nose bleed, nose bleed, nose bleeds, nose bleeds adults, nose bleeds and stress, nose bleeds cause, nose bleeds everyday, nose bleeds in children, nose bleeds in kids, nose bleeds treatment, nose bleeds, nose bleeds, nose bleeds, nose bleeds, nosebleed, nosebleed causes, nosebleed section, nosebleed section, nosebleeds, nosebleeds causes, nosebleeds in adults, nosebleeds in children, nosebleeds in kids, nosebleeds in winter, random nose bleed, what causes nose bleedsm, what causes nosebleeds, what causes nosebleeds, what is epistaxis

**UNITED KINGDOM**

baby nose bleed, baby nosebleed, bad nose bleeds, bleeding nose, cause of nose bleeds, cause of nosebleed, cause of nosebleeds, causes of nose bleeds, causes of nosebleed, causes of nosebleeds, child nose bleed, constant nose bleeds, constant nosebleeds, define epistaxis, epistasis definition, epistasis, epistaxis, epistaxis definition, epistaxis management, epistaxis meaning, epistaxis nhs, epistaxis patient uk, epistaxis treatment, frequent nose bleeds, frequent nosebleeds, headache and nose bleed, headache and nose bleeds, heavy nose bleed, hht, high blood pressure nose bleed, how to stop a nose bleed, how to stop a nosebleed, how to stop nose bleed, how to stop nose bleeds, how to stop nosebleeds, how to treat a nosebleed, keep getting nose bleeds, keep getting nosebleeds, nasal packing, naseptin, nhs nosebleed, nhs nosebleeds, nose bleed, nose bleed cause, nose bleed causes, nose bleed in children, nose bleed nhs, nose bleed what to do, nose bleed, nose bleeding, nose bleeds, nose bleeds adults, nose bleeds cause, nose bleeds children, nose bleeds everyday, nose bleeds in children, nose bleeds in pregnancy, nose bleeds nhs, nose bleeds, nosebleed, nosebleed and headache, nosebleed cause, nosebleed causes, nosebleed in children, nosebleed pregnancy, nosebleed treatment, nosebleed, nosebleeds, nosebleeds and headaches, nosebleeds at night, nosebleeds causes, nosebleeds causes, nosebleeds children, nosebleeds in adults, nosebleeds in children, nosebleeds in children, nosebleeds kids, nosebleeds pregnancy, nosebleeds stress, nosebleeds, nosebleeds, posterior nosebleed, random nose bleed, random nose bleeds, regular nose bleeds, regular nosebleeds, severe nose bleeds, stop a nosebleed, stop nose bleed, stress nosebleed, stress nosebleeds, sudden nose bleed, sudden nosebleeds, what causes a nosebleed, what causes nose bleed, what causes nose bleeds, what causes nosebleed, what causes nosebleeds, what is epistaxis

**NEW ZEALAND**

Nosebleed, nose bleed, nosebleeds, nose bleeds, epistaxis

**GERMANY**

epistaxis, epitaxie, erkaeltung nasenbluten, haeufiges nasenbluten, kleinkind nasenbluten, kopfschmerzen und nasenbluten, nachts nasenbluten, nase blutet, nase blutet innen, nasenbluten, nasenbluten baby, nasenbluten bei kindern, nasenbluten bluthochdruck, nasenbluten erkaeltung, nasenbluten kind, nasenbluten kinder, nasenbluten kopfschmerzen, nasenbluten nasenspray, nasenbluten schnupfen, nasenbluten stoppen, nasenbluten ursachen, nasenbluten was tun, nasenbluten, oft nasenbluten, ploetzliches nasenbluten, schnupfen nasenbluten, staendig nasenbluten, starkes nasenbluten, ursache nasenbluten, ursachen nasenbluten, was tun bei nasenbluten

**NORWAY**

Epistaxis, neseblod, neseblod kreft, neseblod stress, plutselig neseblod

**ITALY**

cause sangue dal naso, come fermare il sangue dal naso, come fermare il sangue, come fermare sangue dal naso, epistas, epistasi, epistassi, epistassi al naso, epistassi bambini cause, epistassi bambini, epistassi cause, epistassi dal naso, epistassi farmaci, epistassi in gravidanza, epistassi nasale, epistassi naso, epistassi nei bambini, epistassi notturna, epistassi rimedi, epistassi significato, epistassi, epitassi, esce sangue dal naso, fermare sangue dal naso, mal di testa e sangue dal naso, perché esce sangue dal naso, perdita di sangue dal naso, perdita sangue dal naso, raffreddore sangue dal naso, sangue da naso, sangue da naso cause, sangue dal naso cosa fare, sangue dal naso di notte, sangue dal naso gravidanza, sangue dal naso pressione, sangue dal naso rimedi, sangue dal naso
